# Life Cycle Assessment of a Thermal Recycling Process as an Alternative to Existing CFRP and GFRP Composite Wastes Management Options

**DOI:** 10.3390/polym13244430

**Published:** 2021-12-17

**Authors:** Sankar Karuppannan Gopalraj, Ivan Deviatkin, Mika Horttanainen, Timo Kärki

**Affiliations:** 1 Fiber Composite Laboratory, Department of Mechanical Engineering, LUT University, P.O. Box 20, 53850 Lappeenranta, Finland; timo.karki@lut.fi; 2 Department of Sustainability Science, LUT University, P.O. Box 20, 53850 Lappeenranta, Finland; ivan.deviatkin@lut.fi (I.D.); mika.horttanainen@lut.fi (M.H.)

**Keywords:** life cycle assessment, composite recycling, carbon fibre, glass fibre, waste disposal, thermal recycling

## Abstract

There are forecasts for the exponential increase in the generation of carbon fibre-reinforced polymer (CFRP) and glass fibre-reinforced polymer (GFRP) composite wastes containing valuable carbon and glass fibres. The recent adoption of these composites in wind turbines and aeroplanes has increased the amount of end-of-life waste from these applications. By adequately closing the life cycle loop, these enormous volumes of waste can partly satisfy the global demand for their virgin counterparts. Therefore, there is a need to properly dispose these composite wastes, with material recovery being the final target, thanks to the strict EU regulations for promoting recycling and reusing as the highest priorities in waste disposal options. In addition, the hefty taxation has almost brought about an end to landfills. These government regulations towards properly recycling these composite wastes have changed the industries’ attitudes toward sustainable disposal approaches, and life cycle assessment (LCA) plays a vital role in this transition phase. This LCA study uses climate change results and fossil fuel consumptions to study the environmental impacts of a thermal recycling route to recycle and remanufacture CFRP and GFRP wastes into recycled rCFRP and rGFRP composites. Additionally, a comprehensive analysis was performed comparing with the traditional waste management options such as landfill, incineration with energy recovery and feedstock for cement kiln. Overall, the LCA results were favourable for CFRP wastes to be recycled using the thermal recycling route with lower environmental impacts. However, this contradicts GFRP wastes in which using them as feedstock in cement kiln production displayed more reduced environmental impacts than those thermally recycled to substitute virgin composite production.

## 1. Introduction

### 1.1. Background

Carbon fibre-reinforced polymer (CFRP) and glass fibre-reinforced polymer (GFRP) composites have been used in high-performance and lightweight applications such as renewable energy, automobiles, construction, aeronautics, aerospace, sports, and defence. The composite’s incredible mechanical properties, especially, strength to weight ratio, attracted industries to utilise these composites in enormous quantities despite their hefty price range [1]. In 2020, the global composite market size reached USD 95.89 billion. By 2027, it is estimated to be at USD 160.54 billion. The majority of the shares were contributed by CFRP and GFRP composites in lightweight applications [2]. In particular, two applications, namely wind turbines (WTs) and aeroplanes, are notable for using CFRP and GFRP composites in higher volumes. Based on the 2021 report by the global wind energy council (GWEC) [3], it is necessary to achieve net-zero carbon dioxide emissions by 2050 in order to avoid climate change, and wind energy plays a significant role. Despite the impressive targets in the year 2020, the report suggests three times more WT installation requirements in the following years to reach such global targets.

Meanwhile, the cumulating end-of-life (EoL) WTs from 1980 need to be disposed of. As the new WTs and aeroplanes utilised a higher volume of CFRP and GFRP composites, they will be available for disposal after 25 years of usage. Overall, an estimation of 1 million tonnes of composite wastes will be generated by 2050 [4]. In these composites, the virgin carbon fibre (vCF) production is recognised as one of the most energy-consuming processes. It consumes 14 times more energy than conventional steel production [5]. However, it is indicated that replacing polyacrylonitrile (PAN) (commonly used precursor) with lignin can significantly reduce the environmental impacts and energy consumption to a certain extent [6]. However, the wastes have to be carefully recycled to reduce virgin fibre production. As the recycled carbon fibres (rCFs) have a higher potential to be reused in automobile industries. They have lower environmental impacts compared with materials typically used in automobile components, namely virgin (v) CFRP, aluminium, and conventional steel [7]. The virgin glass fibre (vGF) production is not as energy-intensive as CF production but is used in applications as GFRP composites in a heavier volume than CFRP composites [8]. However, the GFRP wastes are mainly used as feedstocks in cement production [9].

Earlier, there were no sustainable waste disposal methods studied and established. These composite wastes were typically landfilled and incinerated (energy recovery). After 2008, when the European Waste Frame Directive (2008/98/EC) [10] came into force, it initiated a benchmark on the waste disposal hierarchy, encouraging recycling as an exclusive waste disposal route. As a result, the landfilling taxation was increased. The cost of landfill in the UK stands at GBP 130–140 per tonne. Meanwhile, countries such as Germany and other EU states predominantly ban landfills and have promoted a circular economy in recent times [9].

### 1.2. Literature Review

Various studies have previously used life cycle assessment (LCA) to investigate the environmental effects of various disposal methods concerning CFRP and GFRP composite wastes. Witik et al. (2013) [11] studied three possible disposal methods for CFRP wastes, namely landfilling, incineration (energy recovery), and pyrolysis to recycle CFs (material recovery). They highlighted the advantages of replacing vCFs and vGFs with rCFs as it possesses similar mechanical properties and consumes less energy to recycle using pyrolysis than producing new fibres. However, the authors have assumed values for pyrolysis from various patents and reports making it highly uncertain. La rosa et al. (2016) [12] investigated a chemical recycling method using acetic acid to recycle CFRP wastes. Subsequently, the study used LCA methods, and highlighted that rCFs could substitute vCFs. However, the study failed to address the environmental effects of the hazardous chemicals used in the recycling process on an industrial scale. Plus, the fact that rCFs should be further treated to be reused (system boundary limitation). Li et al. (2016) [13] briefly studied mechanical recycling additional to landfilling and incineration. The study indicates that mechanical recycling is never a suitable CFRP waste disposal approach as the process is costly, 3000 EUR/tonne and does not have proper energy and material recovery paths. 

Meng et al. (2017) [14] studied the fluidised bed process (FBP) to recycle CFRP wastes, and highlighted that recycling consumes 32–50% less energy and reduces 33–51% GWP compared with vCF production. Moreover, the recycling process has lower environmental and economical effects compared with traditional landfilling and incineration routes. However, the processes possess short and randomly oriented rCFs, limiting their reusing ability in various applications (substitution limitation). Subsequently, Meng et al. (2018) [15] investigated mechanical recycling, thermal recycling—pyrolysis and FBP and chemical recycling disposal methods. Overall, the study highlighted recycling CFRP wastes to recover the valuable carbon fibres from being the only sustainable alternative to landfilling and incineration. The LCA results—GWP −19 to −27 kg CO_2_-eq and PED −395 to −520 MJ/kg—indicate a substantial reduction in the environmental impacts. Later Meng et al. (2018) [16] continued the studies addressing the industrial-scale FBP, including the rCF’s fibre alignment (drawbacks from the previous study). The study highlighted that only 15% of the energy from vCF production is required to operate an industrial-scale FBP with an overall recycling cost of 4.31 EUR/kg. Despite these well-established studies in FBP, a functional industrial-scale process has not been established. 

The increase in the popularity of solvolysis at supercritical and subcritical conditions has also reflected an LCA-based study. Vo Dong et al. (2018) [17] briefly investigated the CFRP waste disposal method’s economic and environmental aspects. They studied traditional methods such as landfilling, incineration and material recovery method—mechanical, thermal and solvolysis using supercritical water (SCW)—recycling processes. The study highlights the advantages of using thermal recycling and solvolysis waste management methods using the GWP indicator. Despite their higher price range than landfilling and incineration, the rCFs can substitute vCFs and vGFs with minimum environmental effects. However, there are many practical difficulties in establishing an industrial-scale solvolysis plant. Khalil (2018) [18] performed an in-depth LCA study and developed gate-to-gate recycling models keeping pyrolysis as baseline and solvolysis as an alternative approach. Overall, based on nine impact categories, the study concluded that pyrolysis possesses lower environmental and human health-based impacts compared with solvolysis to recycle CFRP wastes. Pillain et al. (2019) [19] performed a comparative LCA study. They analysed the sustainability aspects and highlighted the advantages of recycling the CFRP wastes by solvolysis using SCW parallel to pyrolysis, electrodynamic fragmentation (mechanical recycling) and pointed out the disadvantages of traditional disposal methods such as incineration and landfill. Similar to the LCA studies, Liu et al. (2019) [20] followed an ecoaudit approach utilising energy consumption to measure the environmental impacts to compare EoL options for WT blades. The study highlighted the solvolysis approach as the future of CFRP and GFRP waste management options for EoL WT applications.

A recent study by Meng et al. (2020) [21] utilised LCA to investigate the possibility to replace vGFs with aligned rCFs in aviation applications. As 500,000 tonnes of EoL aeroplane are expected by 2050, recycling the CFRP wastes using FBP and replacing the vGFs in the aviation application promotes closed-loop recycling with a higher reduction in environmental and economical aspects. However, Tapper et al. (2020) [22] reviewed the LCA studies to analyse the closed-loop CFRP waste disposal methods and pointed out pyrolysis as preferred to FBP in recycling CFRP wastes thermally. Furthermore, La Rosa et al. (2021) [23] studied both closed-loop and open-loop using LCA for recycling CFRP wastes using chemical recycling, and highlighted the advantages of the open-loop approach being more realistic and cost-efficient.

Overall, specific research gaps were noticed from the literature review. It can be seen that the majority of the LCA studies focused only on CFRP waste disposal options. Even though certain studies include discussions about substituting vGFs with rCFs. There are no significant LCA studies dedicated to analysing the GFRP waste disposal methods, as the modern recycling (material recovery) methods for CFRP wastes are similar to GFRP waste. Additionally, most LCA studies involving CFRP waste disposal possess inventory analysis taken from other studies and reports. Only a few works of literature have performed LCA studies for their personally developed recycling methods. 

This study primarily aims to perform an LCA assessment on a recycling route that thermally recycles the CFRP and GFRP wastes and remanufactures the recycled fibres into recycled (r) CFRP and rGFRP composites employing a fresh epoxy resin system using a compression moulding process. Furthermore, LCA was performed over three traditionally practised waste management options: landfilling, incineration, and feedstock in a cement kiln (co-incineration). Finally, a comprehensive LCA assessment was conducted by comparing the LCA results using Global Warming Potential (GWP) and Abiotic Depletion Potential for fossil fuels (ADP_f_) indicators to investigate the feasibility of the thermal recycling process as an alternative to the traditional waste management options. Overall, this study will provide insights into the waste management options of CFRP and GFRP wastes, with material recovery being the highest priority to close the life cycle loop of the composites and encourage a circular economy.

## 2. Methodology

### 2.1. Studied Waste

In this study, manufacturing wastes (pre-consumer) of CFRP and GFRP composites from domestic applications were subjected to study. The wastes were provided by Excel composites Oyj (Heinävaara, Finland). The CFRP wastes with a density of 1.81 g/cm^3^ possessed 55.5 wt% of unidirectional vCF and 44.5 wt% cured epoxy resin. The GFRP wastes with a density of 1.52 g/cm^3^ possessed a 44 wt% laminated thin-ply vGF structure and 56 wt% unsaturated polyester resin (UPR). Apart from the mentioned features, the mechanical properties of the composites were unknown. Overall, it was assumed that the share of carbon fibres and glass fibres in the composites was 55 wt% for this study. The rest was matrix polymers–epoxy resin in the case of CFRP and UPR in the case of GFRP. Furthermore, these composite wastes were recycled and remanufactured to produce rCFRP and rGFRP composites employing fresh resins with the rCFs and recycled glass fibres (rGFs) to close their life cycle loop.

### 2.2. Thermal Recycling Route

**Figure 1:**
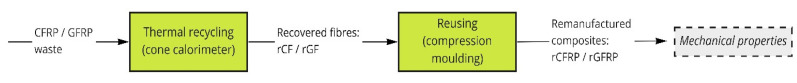
Performed thermal recycling route (modified from [24]).

Figure 1 presents the overall thermal recycling route. The thermal recycling process used for this LCA study is from the author’s previous research work [24]. The process involves incineration and combustion principles using heat radiation in a controlled environment. The developed process is capable of recycling both CFRP and GFRP wastes. The process uses heat flux at 50 kW/m^2^ to recycle these composite wastes separately in an open chamber batch reactor in the presence of air. At 550 °C in atmospheric pressure, the epoxy resin from CFRP wastes was completely evaporated in 20–25 min, leaving clean rCFs in the reactor. Similarly, the UPR from GFRP wastes evaporated in 25–30 min, leaving clean rGFs. The evidence for maximum resin removal from the recycled fibre’s surface was investigated by employing a scanning electron microscope.

The recycled fibres, both rCFs and rGFs, possessed a unidirectional (0), long (105 ± 2 mm) and continuous (end-to-end) fibre arrangement. These fibres were reused by reinforcing with fresh laminating epoxy resin and hardener in a 2:1 ratio using compression moulding. The newly produced composites were further experimentally [24] and numerically [25] examined. Table 1 presents the experimental mechanical properties measured from the produced composites. As seen, two types for each composite were produced based on the fibre (V^f^) and resin volume fraction (V^r^). Overall, the process is capable of recycling CFRP and GFRP composite wastes with a fibre recovery rate of 95–98 wt% for rCFs and 80–82 wt% for rGFs.

### 2.3. Life Cycle Assessment

LCA methodology was primarily performed based on the ISO 14040 [26] and ISO 14044 [27] standards to investigate the respective impacts in various disposal routes. The LCA was modelled using GaBi software (version 9.0.0.42, DP service pack 38) provided by Sphera Solutions GmbH, Leinfelden-Echterdingen, Germany [28].

#### 2.3.1. Goal and Scope Definition

The goal of this LCA study was to model the climate change impacts and depletion potential of abiotic resources of the developed thermal recycling process for CFRP and GFRP waste. Furthermore, the study compares the impacts of this process with other traditional waste disposal methods practised for CFRP and GFRP wastes, namely landfilling, incineration with energy recovery, and the use as feedstock in the cement production. The function of the studied product system is to treat or dispose of CFRP and GFRP wastes. Therefore, the functional unit is 1 kg of CFRP waste and 1 kg of GFRP waste collected in Finland. The study utilises a gate-to-grave (or a bin-to-grave) approach: the assessment of the environmental impacts started from the point of waste generation, thus accounting for its transportation to the treatment facility and ended with the waste being treated. The system expansion approach was utilised to account for the multifunctionality of the studied product system with several scenarios.

**Figure 2:**
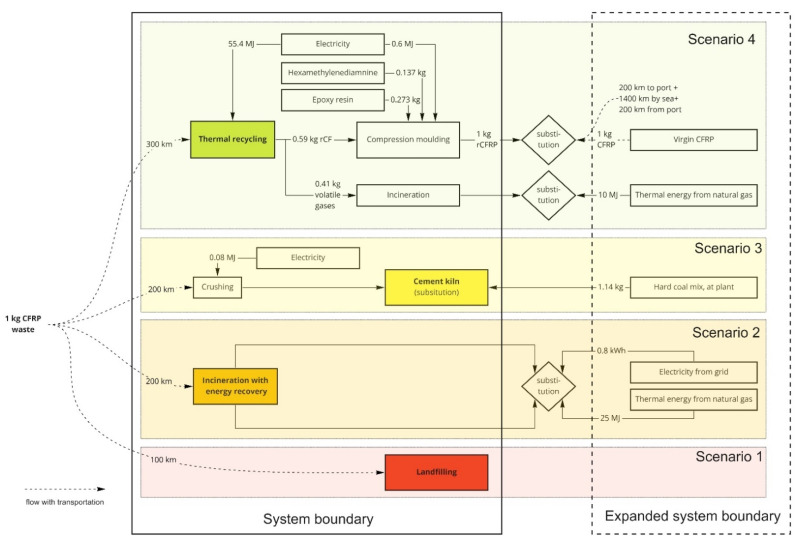
System boundaries of the studied and expanded systems for CFRP waste management.

**Figure 3:**
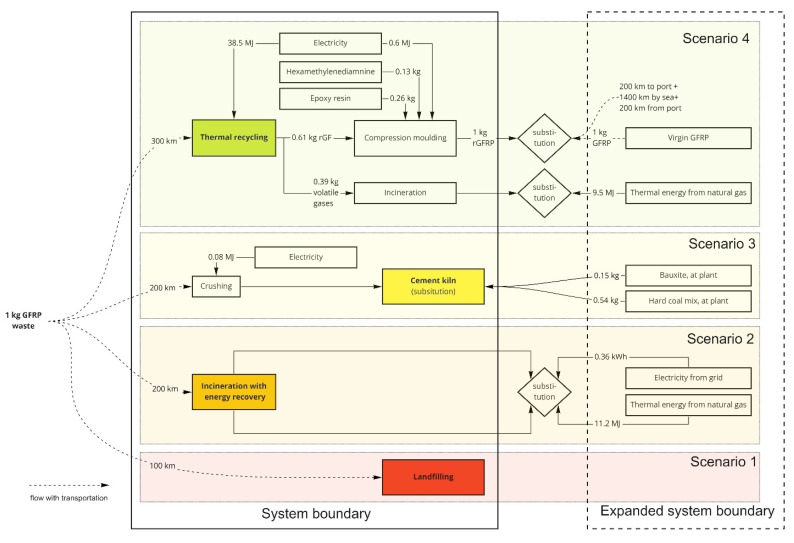
System boundaries of the studied and expanded systems for GFRP waste management.

Figure 2 and Figure 3 presents the system boundaries and expanded systems for CFRP and GFRP waste’s disposal scenarios. The scenarios are placed in the descending order of the EU regulations [10] for waste hierarchy, where recycling is the highest priority. The CFRP and GFRP wastes were considered carrying no burdens from their previous life cycle stages, i.e., so-called “zero burden” approach. For the CF recycling sector, GWP was considered an essential LCA indicator [29]. The life cycle impact assessment was performed for the GWP and ADP_f_ using the characterisation method of the product environmental footprint implemented in GaBi software as “EF 2.0 (Environmental Footprint 2.0)” [30].

The four scenarios concerning this product system were the thermal recycling process (scenario 4) from the composite wastes to rCFRP and rGFRP production. It consists of two system expansions, first to substitute with virgin composite production and the thermal energy produced during recycling to substitute with natural gas. Cement kiln production (scenario 3) involves hard coal mix for both the composites and an additional bauxite mix for GFRP wastes. The incineration (scenario 2) process utilises energy recovery and an expanded system for electricity and thermal energy substitutions. Finally, landfilling (scenario 1) is used without any possible substitutions.

#### 2.3.2. Life Cycle Inventory

Thermal recycling of composite waste: The data on thermal recycling of CFRP and GFRP wastes were obtained from the laboratory-scale equipment. Recycling 1 kg of CFRP wastes consumed 15.4 kWh of electricity under stabilised process conditions and yielded 0.59 kg of rCFs. Recycling 1 kg of GFRP wastes consumed less electricity than CFRP wastes, 10.7 kWh, and yielded 0.61 kg of rGFs. The rest of the resin system’s mass in the composite wastes was gasified and removed from the system. The gases were not captured in the laboratory conditions but were expected to happen on an industrial scale to regenerate the heat within the system. Therefore, the incineration of the gases was modelled in this study. The process was modelled using the unit process “FI: Plastics (unspecified) in waste incineration plant”. This process is expected to represent the impact from incinerating the resin system vaporised during the process. The thermal energy generated during the incineration process was substituted with thermal energy from natural gas using the process “FI: Thermal energy from natural gas”.

Once the fibres (rCFs and rGFs) were recycled, they were used to produce rCFRP and rGFRP composites. These recycled fibres were mixed with laminating epoxy resin and hardener in a 2:1 ratio. The produced recycled composites were assumed to have 60 wt% recycled fibre volume fraction and 40 wt% epoxy resin. A low energy-consuming compression moulding technique was used to produce the new composites. The process required 0.167 kWh to produce 1 kg of rCFRP or rGFRP. The process used for epoxy production was “DE: Epoxy resin (EP) mix”. The hardener was modelled using the process “EU-28: Hexamethylenediamine (HMDA); from butadiene via adiponitrile”. The produced rCFRP and rGFRP were compared with the virgin polymers.

Production of virgin CFRP: To produce vCFRP, CFs needed to be produced first. Their production was modelled using the data reported elsewhere [5,31]. The inventory of the production process and the unit processes used are presented in Table 2. 

The CFRP composite’s composition was assumed in this study, corresponding to the results of the thermal recycling experiments. Thermal recycling of CFRP yielded 59% of rCF. However, a part of this mass is epoxy resin which did not gasify. Therefore, in this study, the share of carbon fibres in virgin CFRP was assumed to be 55 wt%. The rest of the mass was epoxy resin and hardener mixed in the ratio of 2:1, as is the case with the rCFRP production. The CFRP production was modelled using the low-pressure resing transfer moulding (LPRTM) process. Vita et al. [32] reported the energy consumption of the LPRTM process for producing one car CFRP hood. The energy consumption of the pre-forming, moulding, mixing, and metering stages of the process equals 2.85 kWh per 1 kg of CFRP produced.

Production of virgin GFRP: The production of vGFRP was modelled as mixing 0.55 kg of vGFs (“DE: Glass fibres”) with 0.45 kg UPR (“DE: Polyester resin (unsaturated) (UP)”). Electricity consumption was modelled in the same way as in the production of the CFRP, which is equal to 2.85 kWh [32].

Use of composite waste in a cement kiln: Before the co-incineration of CFRP and GFRP wastes in a cement kiln, the waste is crushed to ensure suitable particle size. The electricity consumption of waste crushing was taken from the study by Witik et al. [11] and equalled 0.09 MJ per 1 kg of crushed composite waste. In the cement kiln, both composite wastes were assumed to substitute coal. The substitution was calculated based on the energy content of composite waste. The higher heating value (HHV) of CFRP was 32 MJ/kg, and the HHV of coal was 28 MJ/kg, so the mass of coal substituted with 1 kg of CFRP waste was 1.14 kg. 

In the case of GFRP, the calculation took into account only the HHV of UPR since GFs are mineral in nature. The HHV of UPR was 33.5 MJ/kg, and accounting for its share of 45% in the GFRP waste, the mass of substituted coal was 0.54 kg. At the same time, GFs were assumed to substitute bauxite due to their high aluminium content. The aluminium oxide content in E-glass fibre is 8%, and that in bauxite is 50%. Therefore, 1 kg of GFRP waste can substitute 0.15 kg of bauxite in the process. The emissions from the cement kiln were modelled based on the carbon content of the fuels: 92% for CFRP, 61.4% for UPR, and 65% for coal. The substituted products were modelled using the following processes: coal—“FI: Hard coal mix”, and bauxite—“EU-28: Bauxite”. Coal transportation was included in the unit process, whereas bauxite was reported in a previous study by Khan et al. [33].

Incineration of composite waste: The incineration of CFRP and GFRP waste was modelled using available unit processes. The incineration of CFRP waste was modelled using the process “EU-28: Plastic (unspecified) in waste incineration plant” since its heating value and composition are similar to plastics. The incineration of GFRP was modelled according to the content of GFs and UPR. The GFs incineration was modelled using the process “FI: Inert material in waste incineration plant”, whereas incineration of UPR was modelled in the same way as for the CFRP waste. The efficiencies of heat and electricity generation were adjusted to represent the specific condition of Nordic countries: 9.6% for electricity generation and 82.9% for heat generation.

Landfilling of composite waste: Landfilling of CFRP and GFRP wastes were modelled in the same way, unlike in the case of incineration because both waste types are not biodegradable are expected to behave similarly in the landfill. Landfilling was modelled using the process “EU-28: Plastic waste on landfill”.

Transportation: Table 3 shows the distances and transportation modes used in the study. The distance to the disposal facilities was assumed based on their availability: landfills are the most common disposal sites, so they have the shortest distance, whereas a thermal recycling facility would be the most scarcely located.

#### 2.3.3. Sensitivity Analysis

The sensitivity analysis was performed for the composites by varying the parameters concerning electricity consumption involved in the production process and the product substitution. Amongst the impacts to produce rCFRP and rGFRP composite, the electricity consumption to thermally recycle the composite wastes is expected to be high and will significantly influence the impacts. In CFRP composites, the possibility to replace 100% of vCFRP with rCFRP (1:1) is practically not possible. Therefore, various possible ratios were taken under consideration. Similarly, in GFRP composites, the vGF production is less energy-intensive than producing rGF and vCF. Hence, the possibilities in reducing the energy consumption to produce rGF were taken under consideration. Overall, the sensitivity analysis was conducted using break-even point analysis.

## 3. Results

### 3.1. Impacts from Recycled Composite Production

#### 3.1.1. rCFRP Production

**Figure 4:**
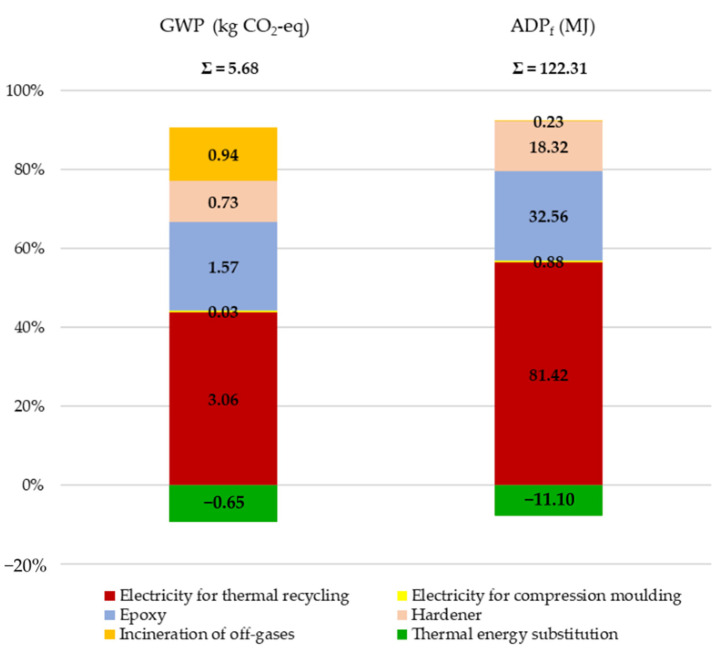
GWP and ADP_f_ results for producing 1 kg of rCFRP composites.

Figure 4 presents the GWP and ADP_f_ impacts from thermally recycling the CFRP wastes and rCFRP production. As seen, the overall carbon footprint of producing 1 kg of rCFRP was 5.68 kg CO_2_-eq. The largest share of impact 3.07 kg CO_2_-eq (53.87%) came from electricity consumption to generate the designated heat flux of 50 kW/m^2^ in order to evaporate the epoxy resin from the composite wastes. The second-largest contribution of 2.30 kg CO_2_-eq (40.5%) was from the production of epoxy and hardener (resin system) to be employed to produce rCFRP composites. Electricity consumed by compression moulding possessed a minor impact of 0.03 kg CO_2_-eq (0.53%) on the results. Finally, the incineration of exhaust fumes from thermal recycling generated was 0.94 CO_2_-eq. (16.55%). However, it was substituted by energy produced from natural gas, whose production emits 0.65 CO_2_-eq. The ADP_f_ results to produce 1 kg of rCFRP composites were 122.31 MJ. Similar to GWP results, most of its impact, 81.42 MJ (66.56%), comes from the electricity involved in the recycling process. Subsequently, the resin system holds 50.88 MJ (41.60%). Finally, the lower impact contributions were from electricity consumption in compression moulding at 0.88 MJ (0.72%) and the incineration of exhaust fumes at 0.23 MJ (0.19%). The substituted energy produced from natural gas was 11.10 MJ (9.07%).

#### 3.1.2. rGFRP Production

**Figure 5:**
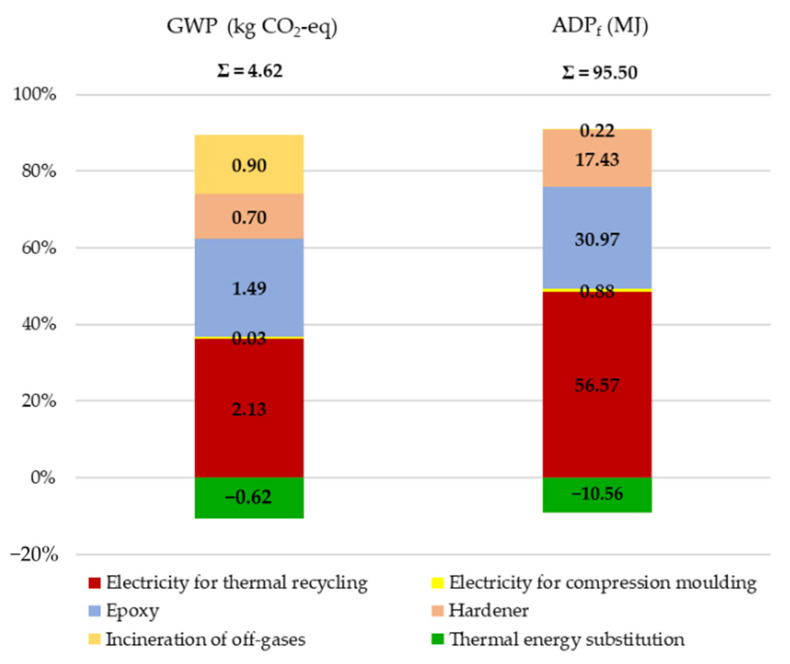
GWP and ADP_f_ results for producing 1 kg of rGFRP composites.

Figure 5 presents the GWP and ADP_f_ impacts from thermally recycling the GFRP wastes and rGFRP production. As seen, the overall carbon footprint to produce 1 kg of rGFRP composites was 4.62 kg CO_2_-eq. The maximum impact was from the epoxy and hardener (resin system) production 2.19 kg CO_2_-eq (47.40%). At the same time, the electricity consumption in the thermal recycling process possesses similar emissions of 2.13 kg CO_2_-eq (46.10%). The emissions from the incineration of exhaust fumes from the recycling process were 0.94 kg CO_2_-eq (20.34%) and were substituted by 0.65 kg CO_2_-eq of energy produced by the natural gas. Finally, the minimum emissions were 0.03 kg CO_2_-eq (0.65) electricity consumed by compression moulding. The ADP_f_ results to produce 1 kg of rGFRP composites were 95.50 MJ. The electricity consumption during the recycling process possess the maximum impact of 56.57 MJ, and impacts from resin system production were 48.40 MJ. The percentage of contribution for both these impacts was 59.23% and 50.68%. The incineration of off-gases and electricity to compression moulding holds the lowest impacts at 0.22 MJ and 0.88 MJ. At the same time, the gas emissions were substituted with 10.56 MJ energy produced from natural gas.

### 3.2. Environmental Impacts: Gate-to-Grave

#### 3.2.1. CFRP Waste Disposal Methods

**Figure 6:**
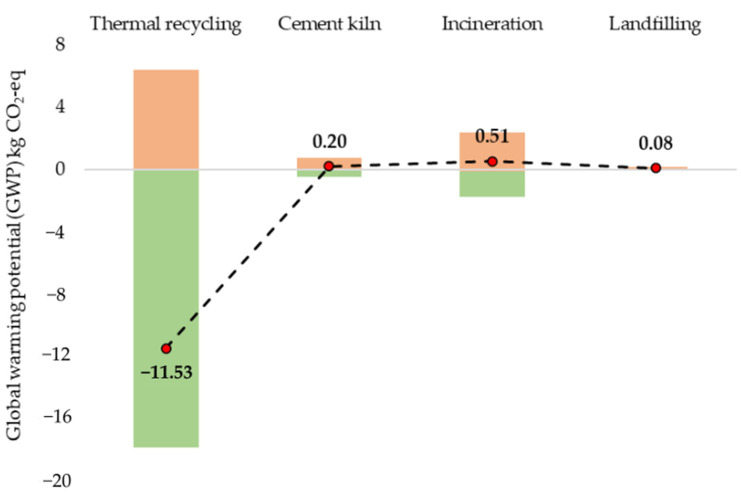
GWP of various CFRP waste disposal scenarios.

Figure 6 presents the GWP results for 1 kg of CFRP waste disposal using thermal recycling, cement kiln, incineration, and landfill. As seen, the thermal recycling route appeared to have an immense potential to reduce the impacts of climate change and preserve fossil resources compared with other disposals scenarios. Such high reduction potentials were enabled through the substitutions from vCFRP production. The production of 1 kg of vCFRP has a GWP of 17.20 kg CO_2_-eq. The impacts from producing vCF alone hold 14.11 kg CO_2_-eq (82.03%), and the remaining impacts were contributed from the epoxy resin system and electricity for the resin transfer moulding process. Therefore, producing 1 kg of rCFRP reduces the overall GWP by 11.53 kg CO_2_-eq, including the direct emissions from recycling and the avoided impact. 

The GWP of the other disposal scenarios were relatively close, ranging at 0.08–0.51 kg CO_2_-eq. Amongst which landfilling has the lowest impact of 0.08 kg CO_2_-eq per 1 kg of CFRP waste disposal. No landfill gas was generated. The emissions were from the working machinery in landfills. The emissions from incineration were 2.30 kg CO_2_-eq. It was almost twice higher than the 0.94 kg CO_2_-eq emissions from incinerating exhaust fumes generated during the thermal recycling. The energy recovered from incineration could substitute 1.80 kg CO_2_-eq in electricity and thermal energy but completely burn the CFRP waste leaving only ashes behind. Finally, the cement kiln route possessed 0.20 kg CO_2_-eq emissions with 0.47 kg CO_2_-eq hard coal substitution. Overall CFRP waste disposal route via thermal recycling seems to be more efficient and sustainable than other disposal scenarios. Based on the sensitivity analysis, the rCFRP composites should be capable of replacing ≥ 30% vCFRP composites, as the overall GWP will be 0.51 kg CO_2_-eq, equal to the impacts from the incineration disposal route (non-sustainable). Any substitution ratio < 30% will result in higher impacts compared with other traditional disposal routes. 

**Figure 7:**
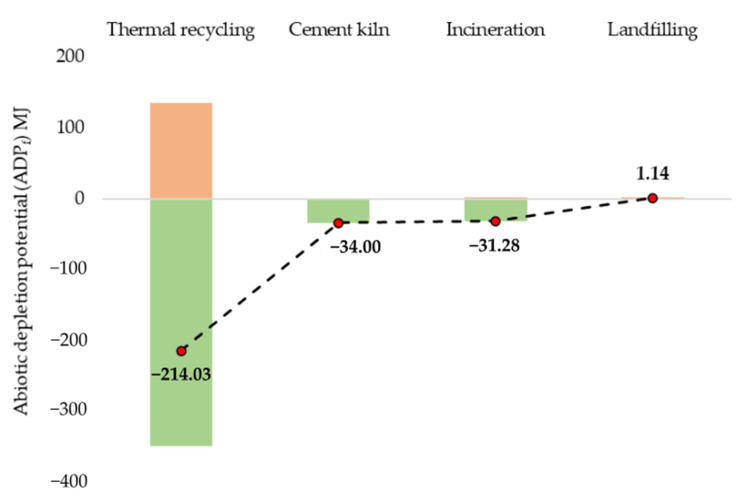
ADP_f_ of various CFRP waste disposal scenarios.

Figure 7 presents the ADP_f_ results for 1 kg of CFRP wastes disposal using various scenarios. Similar to GWP results of the thermal recycling route, the ADP_f_ is reduced by 214 MJ per 1 kg of rCFRP, primarily due to avoided production of vCF from substitutions. After thermal recycling, cement kiln’s hard coal substitution have reduced the impacts by 34 MJ. Similarly, the energy substitutions from incineration have reduced impacts by 31.28 MJ. Landfilling impacts were 1.14 MJ without any possibility for substitution.

#### 3.2.2. GFRP Waste Disposal Methods

**Figure 8:**
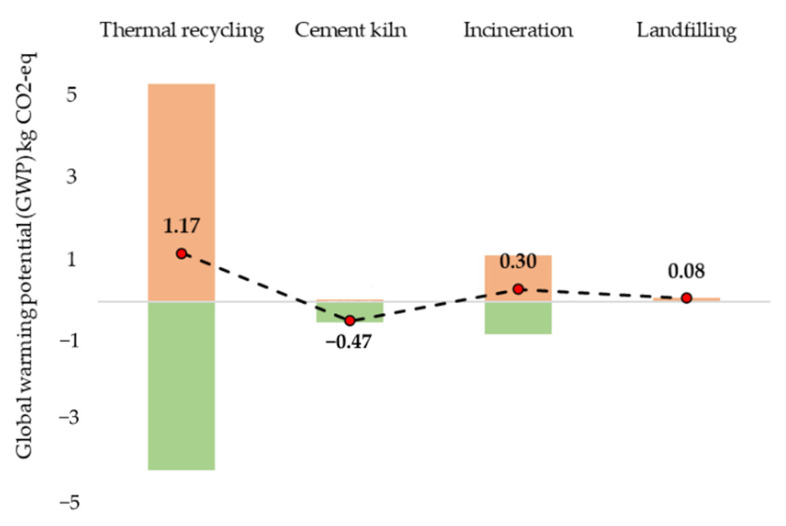
GWP of various GFRP waste disposal scenarios.

Figure 8 presents the GWP results for 1 kg of GFRP waste disposal using thermal recycling, cement kiln, incineration and landfill. As seen, the cement kiln possesses a reduced GWP impact of 0.47 kg CO_2_-eq due to GFRP being favourable for cement kiln production along with hard coal and bauxite substitution. The impacts from the thermal recycling route were 1.17 kg CO_2_-eq, including the substitutions from vGFRP production. Despite the 30.71% lower energy consumption in thermal recycling GFRP wastes compared with CFRP wastes, the higher impact values were reflected due to the lower substitution values from vGF production. As the vCF production was highly energy-intensive with 17.20 kg CO_2_-eq, substitutions with rCFRP composites 5.38 kg CO_2_-eq significantly reduced overall emissions, whereas in the case of vGF production, the GWP emissions were 3.43 kg CO_2_-eq compared with the GWP emissions of 4.62 kg CO_2_-eq from producing rGFRP. Overall, 34.70% additional emissions were created by producing rGFRP composites. The emissions from incineration were 1.09 kg CO_2_-eq, whereas the reductions from the energy substitution were 0.80 kg CO_2_-eq. Finally, landfilling with 0.08 kg CO_2_-eq emissions was similar to the landfill emissions from CFRP wastes. 

Based on the sensitivity analysis, reducing 40% of emissions from energy consumption for the thermal recycling process will result in impacts similar to incineration. Reducing ≈ 77% impacts results in emissions similar to the cement kiln route. However, for scenarios such as incineration and cement kiln, when end-of-life WTs are used as GFRP waste sources, high energy-intensive processes such as shredding (size reduction) should be used to reduce the enormous GFRP wastes size from 60–70 m to 5–6 mm. Overall, the cement kiln route seems to be more sustainable than the thermal recycling route. 

**Figure 9:**
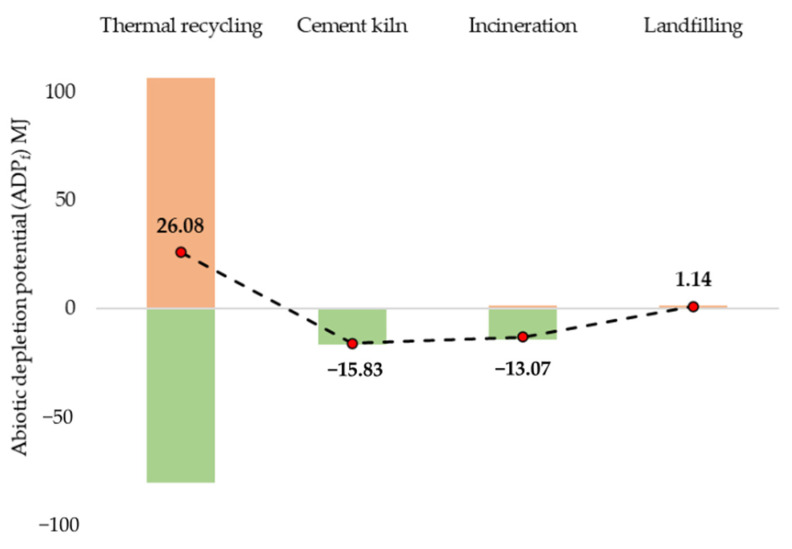
ADP_f_ of various GFRP waste disposal scenarios.

Figure 9 presents the ADP_f_ results for 1 kg of GFRP wastes using various disposal scenarios. As seen, the thermal recycling route displays a higher impact of 26.08 MJ, including the substitutions from vGFRP production with a UPR system. Subsequently, cement kiln 15.83 MJ and incineration 13.07 MJ had comparable reduced impacts after their respective substitutions. The landfill possessed 1.14 MJ. 

## 4. Discussion

The traditional waste disposal routes, namely landfill and incineration (energy recovery), have unfavourable environmental impacts. As mentioned in the literature [1,22,34], the hefty landfill taxation has established waste management industries to avoid landfills altogether. Furthermore, considering the price of vCFRP and vGFRP composites, the popularity of disposal via incineration has also reduced drastically. In particular, the low energy recovery rate with higher emissions and piles of ashes after incineration made it an undesirable disposal route for CFRP and GFRP wastes. The LCA results for CFRP waste disposal via incineration were higher than GFRP wastes. Most CFRP grades are thermal insulators, and heat-based approaches require added effort to react with CFRP composites. Such behaviours are visible even in the adopted thermal recycling route with higher energy consumption to recycle CFRP wastes than GFRP wastes.

The LCA results for CFRP and GFRP waste disposal via cement kiln were expected. The CFRP wastes are not suitable to use as feedstocks in cement production, but perform better than incineration with comparatively lower environmental impacts. On the other hand, the results for GFRP wastes were surprisingly positive compared with the proposed thermal recycling route with material recovery. GFRP composites majorly consist of E-glass made from aluminium borosilicate, calcium-based fillers and resin. When used in the cement kiln, the resin evaporation provides heat, and the minerals are used as feedstocks in cement production. Overall, GFRP can be disposed of without any residues. However, the presence of boron makes it challenging to replace 100% of cement kiln fuel with GFRP wastes but can be used to a certain extent. Moreover, the GFRP wastes must not contain impurities or metals, and the waste should be size reduced to fit in [35,36], which is highly energy consuming when considering EoL WTs with 60–70 m-long components. 

The GWP and ADP_f_ results to produce rCFRP and rGFRP composites highlighted the maximum impacts from energy consumption during the recycling phase. The measured electricity consumption to thermally recycle 1 kg of CFRP wastes was 55.44 MJ, and GFRP wastes were 38.52 MJ. However, considering an upscaling to pilot process and further towards industrial-scale process, the thermal heat produced during the resin evaporated will be regenerated into the system. Hence, reduce the electricity consumption to a certain extend. Additionally, replacing the electricity source with renewable energies will result in maximum sustainable outcomes with lower environmental emissions. Still, at this laboratory scale, it is required to compare the LCA results with currently dominating recycling processes as highlighted from the previous study [1], namely pyrolysis and solvolysis using supercritical/ subcritical water or mild solvents. LCA studies involving pyrolysis had mostly adopted inventory data from industrial-scale pyrolysis operated by ELG carbon fibres (UK), at present named Gen2carbon. The pyrolysis energy consumption was 30 MJ per kg of CFRP waste recycling with a 2000 tonne/year capacity, resulting in GWP 2.88 kg CO_2_-eq [15]. For solvolysis, a recent study by Liu et al. 2021 [37] recycled 1 kg of CFRP wastes via solvolysis using supercritical n-butanol at 49.21 MJ/kg. The LCA study by Nunes et al. 2018 [38] highlighted the energy required to recycle 1 kg of CFRP waste via steam thermolysis to be 54 MJ. Additionally, 17.64 MJ energy was spent to pre-process (cutting) 1 kg of CFRP waste.

Furthermore, various studies have compared the LCA results of pyrolysis and solvolysis. Khalil (2018) [18] highlighted the energy required to recycle 1 kg of CFRP waste via pyrolysis to be 12.53 MJ with 0.96 kg CO_2_-eq GWP and 25 MJ via solvolysis with 16.2 kg CO_2_-eq GWP. However, an additional 145 MJ of energy is required for solvolysis to produce the supercritical state for water (4.67 kg) to recycle 1 kg of CFRP waste. These results have significantly lower environmental impacts compared with the thermal recycling route from this study. However, a significant LCA study by Vo Dong et al. (2018) [17] showed −19 to −22 kg CO_2_-eq GWP impacts to dispose of 1 kg of CFRP waste via pyrolysis and solvolysis SCW, including the substitutions with vCFRP production. In comparison, the results from this study have impacts of −11.53 kg CO_2_-eq GWP, including the substitution from vCFRP production. He et al. (2020) [39] studied pyrolysis and solvolysis to recycle CFRP waste. They focused on the energy demand during pre-recycling and post-recycling phases and highlighted that energy conservation is possible if the recycled carbon fibre’s structural integrity is maintained. Similar to the fibre arrangements from the thermal recycling process [24], resulting rCFs and rGFs were unidirectional, long and continuous. Overall, as noticed from the LCA studies, no significant studies were available to compare the disposal routes of GFRP composites. However, some studies [11,17,21] have proposed rCF to replace vGFs. Such research gaps can be understood from the LCA result of this study as they could be related to the advantages of the cement kiln process (energy recovery), the defects (char formation) from recycling GFRP wastes, and rGFRP’s reduced mechanical properties failing to replace vGFRP composites, even to a minimum extent.

There are certain limitations faced in this study, such as the vCFRP and vGFRP production being considered perfect without any wastage, which is practically not possible. In incineration modelling, CFRP and GFRP wastes were assumed to be plastic, as no proper process was available for those wastes. The End-of-life WTs are enormous and require an energy-intensive mechanical shredding process to reduce the size of the waste. Finally, a 100% fibre recovery rate (yield) was considered from thermally recycling the composite wastes. However, the original fibre recovery rates were 95–98 wt% for rCFs from CFRP wastes and 80–82 wt% for rGFs from GFRP wastes. Despite these limitations, the results obtained from this study seems to be realistic and coherent when compared with the similar LCA studies from the literature.

## 5. Conclusions

This study analysed various waste disposal methods for CFRP and GFRP wastes using the LCA methodology, especially, focusing on a thermal recycling route developed from the previous study [24] to recycle and remanufacture CFRP and GFRP wastes into rCFRP and rGFRP composites. Three more traditional waste disposal scenarios were analysed: landfill, incineration with energy recovery, and feedstock in cement kiln production. The climate change results and fossil fuel consumption for CFRP waste disposal through the thermal recycling route seem sustainable, with a significant potential to substitute vCFRP. When rCFRP replaces vCFRP with a ratio of 1:1, i.e., 100% of recycled composites to virgin composites, the combined GWP emissions will be −11.43 kg CO_2_-eq. Additionally, taking into account the high mechanical properties (>90%) of rCFRP reported in various studies [1,40,41] and lower emissions (3.06 kg CO_2_-eq) from recycling the pricy CFs. The rCFRP substitutions with vCFRP played a significant role in emphasising circular economy by reducing virgin composite production and encouraging the reuse of recycled composites.

The climate change results and fossil fuel consumption for GFRP waste disposal as feedstocks in cement kiln seems sustainable with −0.47 kg CO_2_-eq. These results are better than the proposed thermal recycling process results of 1.17 kg CO_2_-eq, including the substitution with vGFRP production. The high environmental impacts occurred significantly due to the energy consumption (38.52 MJ/kg) to thermally recycle the GFRP wastes. Additionally, vGF production possesses more limited energy consumption, and the benefits from substituting vGFRP with rGFRP composites were not possible. However, material recovery was totally absent despite the positive results of using GFRP wastes as feedstocks in cement kiln production. Incineration and landfill, straightforward and popular disposal options, were strictly unsuitable for treating CFRP and GFRP wastes. The incineration results imply higher environmental impacts with a lower energy recovery rate and zero material recovery possibility.

It is necessary to further study an optimised thermal recycling route with industrial-scale operating conditions, especially for GFRP waste disposal. The possibility to regenerate the heat within the recycling system will result in reducing the energy consumption at the same time, increasing the recycling capacity to satisfy the demands in material recovery and substituting their virgin counterparts.

## Figures and Tables

**Table 1:** Mechanical properties of the produced rCFRP and rGFRP composites [24,25].

Composite Recipes	V^f^ (wt%)	V^r^ (wt%)	Tensile Strength (MPa)	Young Modulus (GPa)	Impact Strength (kJ/m^2^)	Fracture Strain (No Unit)	Density (g/cm^3^)	Poisson Ratio
rCFRP	60 ± 2	40 ± 2	235.70	60.80	53.61	0.00683	1.52	1.52
	40 ± 2	60 ± 2	210.34	45.28	49.98	0.00827	1.64	1.64
rGFRP	60 ± 2	40 ± 2	114.58	30.72	41.05	0.00272	1.77	1.77
	40 ± 2	60 ± 2	65.42	27.37	18.99	0.00156	1.85	1.85

**Table 2:** Life cycle inventory of the carbon fibre production process [5,31].

	Amount	Unit	Unit Process
Inputs			
Amonium bicarbonate	0.02	kg	RER: market for ammonium bicarbonate ecoinvent 3.6
Epoxy resin	0.01	kg	DE: Epoxy resin (EP) mix
Polyacrylonitrile fibres	1.89	kg	EU-28: Polyacrylonitrile Fibres (PAN)
Polydimethylsolixane	0.01	kg	GLO: market for polydimethylsiloxane ecoinvent 3.6
Potassium permanganate	0.1	kg	GLO: market for potassium permanganate ecoinvent 3.6
Sulphuric acid	0.02	kg	EU-28: Sulphuric acid (96%)
Water	2.77	l	EU-28: Process water from surface water
Electricity	20.2	kWh	EU-28: Electricity from grid mix
Heat	98.4	MJ	EU-28: Thermal energy from natural gas
Outputs			
Carbon fibres	1	kg	-
Carbon dioxide	0.63	kg	-
Nitrogen monoxide	0.33	kg	-
Nitrogen dioxide	0.66	kg	-

**Table 3:** Transportation distances and modes.

Flow	from	to	Distance	Transportation Mode
CFRP waste/GFRP waste	Generation place	Recycling facility	300 km	Truck ^1^
		Cement kiln	200 km	Truck ^1^
		Incineration plant	200 km	Truck ^1^
		Landfill	100 km	Truck ^1^
rCFRP/rGFRP	Recycling facility	Customer	100 km	Truck ^1^
vCFRP/vGFRP	Production	Port in Germany	200 km	Truck ^1^
	Port in Germany	Port in Finland	1400 km	Sea-going container ship ^2^
	Port in Finland	Consumer	200 km	Truck ^1^

^1^—GLO: Truck, Euro, 5, 28–32 tonne gross weight/22 tonne payload capacity; ^2^—EU-28: Container ship ocean incl. fuel, 27,500 dwt payload capacity, ocean-going.

## Data Availability

The data presented in this study are available on request from the corresponding author.
